# Supplementary material for: A Meta-Analysis of Observational Studies on Prolactin Levels in Women with Polycystic Ovary Syndrome
Source: Diagnostics (Basel). 2022 Nov 23;12(12):2924. doi: 10.3390/diagnostics12122924 (PMC9777544; doi:10.3390/diagnostics12122924)
Supplement: Supplementary file 1 [file diagnostics-12-02924-s001.zip › diagnostics-2011334-supplementary.pdf]

Supplementary Table S1. Quality assessment of included studies using the Newcastle–Ottawa Quality Assessment Scale for cross-sectional study.

	SELECTION				COMPARABILITY	OUTCOME		Total scores
**Author**	Representativeness of the samples	Sample size	Non-responders	Ascertainment of the exposure (risk factor)	A: study controls for age and/or BMI<br>B: control for any additional factor	Assessment of the outcome<br>a) Independent blind assessment. **<br>b) Record linkage. **<br>c) Self report. *	Statistical test	
Mahboobifard etal, 2021	*	*	*	*	*	**	*	8
Salem et al, 2015	*	*				**	*	5
Yasar et al, 2022	*			*	**	**	*	7
Sahmay et al, 2013		*		*	**	*	*	6
Yang, 2020	*		*	*		*	*	5
Glintborg et al. 2014		*		*	*	**	*	6
Jiang et al, 2021		*	*	*	*	*	*	6
Noroozzadeh et al. 2016	*	*	*	*	*	**	*	8
Yang, 2021		*	*	*	**	**	*	8
Güdücü et al, 2012		*	*	*	*	**	*	7

Supplementary Table S2. Quality assessment of included studies using the Newcastle–Ottawa Quality Assessment Scale for case-control study.

	SELECTION				COMPARABILITY	EXPOSURE			
**Author**	case definition	Representativeness of the cases	Selection of Controls	Definition of Controls	A: study controls for age and/or BMI<br>B: control for any additional factor	Ascertainment of exposure	Same method of ascertainment for cases and controls	Non-Response rate	**Total scores**
Calzada. 2019	*			*	*	*	*		5
Katulski. 2014	*			*	**	*	*		6
Erol, 2017	*			*	*	*	*	*	6
Khashchenko, 2020	*			*	*	*	*	*	6
He et al, 2020	*			*	**	*	*	*	7
Oncul l, 2020	*			*	**	*	*	*	7
Sharif, 2017	*			*	**	*	*	*	7
Panidis, 2005	*			*	*	*	*	*	6
Piouka,2009	*			*	*	*	*	*	6
Yılmaz , 2015	*			*	*	*	*		5
Arpaci,2018	*			*	*	*	*	*	6
Yue, 2018	*			*	*	*	*	*	6
Banaszewska,2020	*			*	**	*	*	*	7
Zohrabi,2017	*			*	**	*	*		6
de-Medeiros,2017	*			*	*		*	*	5
Wang,2008	*			*	*	*	*	*	6
Özcan,2012	*			*	*	*	*	*	6
Rahmani,2018	*			*	**	*	*	*	7
Topcu, 2006	*			*	**	*	*	*	7
Su et al, 2011	*			*	**	*	*	*	7

Supplementary Table S3: Quality assessment of studies included using the Newcastle–Ottawa Quality Assessment Scale for cohort studies

	SELECTION				COMPARABILITY	OUTCOME			Total scores
**Item** / **Author, Year**	Representativeness of the exposed cohort	Selection of the non-exposed cohort	Ascertainment of exposure	No outcome of interest at start of study	A: Study controls for age and/or BMI<br>B: Study controls for other confounders	A: doctor's diagnosis OR objective measurements<br>B: parent/self-reported doctor's diagnosis OR use of medication	Follow-up long enough for outcomes	Adequacy of follow up of cohorts	
Kim,2022		*		*	**	*	*	*	8*
Rashidi,2016		*	*	*	*	*	*	*	7*
